# Intravascular ultrasound-guided reentry wiring with tip-detection technique for chronic total occlusion of lower extremity artery disease

**DOI:** 10.1186/s42155-024-00503-0

**Published:** 2024-12-07

**Authors:** Naoki Hayakawa, Hiromi Miwa, Yasuyuki Tsuchida, Shinya Ichihara, Shunsuke Maruta, Shunichi Kushida

**Affiliations:** Department of Cardiovascular Medicine, Asahi General Hospital, I-1326 Asahi, Chiba, 289-2511 Japan

**Keywords:** Chronic total occlusion, Endovascular therapy, Intravascular ultrasound, Tip-detection

## Abstract

**Background:**

Endovascular therapy is an effective method for revascularization in lower extremity artery disease, but treating chronic total occlusion (CTO) remains challenging. This is particularly true for patients with severe calcification, poor run-off in below-the-knee arteries, or limited access sites, where even guidewire (GW) passage can be difficult and bidirectional approaches are often not feasible. The tip-detection (TD) method has been reported as a useful technique in coronary artery CTO interventions, allowing real-time visualization of the GW tip direction. Here, we applied the TD technique for peripheral CTO intervention.

**Case presentation:**

Case 1 involved a 71-year-old man with a right toe ulcer. Angiography revealed total occlusion from the right anterior tibial artery (ATA) to the proximal dorsalis pedis artery. While attempting IVUS-guided parallel wiring, the GW could not advance through the intraplaque route because of severe calcification. We intentionally advanced the GW and IVUS into the subintimal space of the ATA to bypass the calcified lesion and performed IVUS-guided reentry using the TD technique in the distal ATA, where calcification was less severe. The second GW successfully passed through the intraplaque of the distal ATA and into the true lumen of the dorsalis pedis artery. Case 2 involved a 60-year-old man with bilateral intermittent claudication. Angiography revealed severe stenosis of the right common iliac artery (CIA) and CTO of the left CIA. Because of anatomical limitations and access site challenges, the antegrade approach for the left CIA was unsuccessful, and retrograde intraluminal wiring was difficult because of flexion and calcification. We advanced the GW and IVUS into the subintimal space and performed IVUS-guided reentry using the TD technique to access the true lumen of the proximal CIA. Finally, bilateral VBX stent grafts were implanted using the kissing stent technique.

**Conclusions:**

IVUS-guided reentry wiring with the TD technique may offer a useful solution for passing complex peripheral CTO lesions in cases where only a uni-directional approach is feasible.

**Supplementary Information:**

The online version contains supplementary material available at 10.1186/s42155-024-00503-0.

## Background

Endovascular therapy (EVT) is a widely used method of revascularization for patients with lower extremity artery disease [1]. Although the success rate of EVT for chronic total occlusion (CTO) lesions has improved with the introduction of various retrograde approaches and reentry devices, there are still cases in which the guidewire (GW) cannot pass [2–4]. We previously reported the use of an intravascular ultrasound (IVUS)-guided parallel wiring technique for complex BTK CTO lesions, known as the AnteOwl WR^®^ intravascular ultrasound-guided parallel wiring to a BTK artery (EXCAVATOR) technique, to overcome these challenges [5, 6]. However, passing the GW through all CTOs intraplaque has sometimes proven difficult. Okamura et al. [7] recently introduced a tip-detection antegrade dissection reentry (TD-ADR) technique using the AnteOwl WR^®^ IVUS device (Terumo, Tokyo, Japan) (hereafter referred to as AnteOwl) for coronary artery CTO interventions [7, 8]. This technique employs the AnteOwl to guide the GW tip in real time, allowing operators to accurately direct the GW toward the target. The TD method enables precise reentry into the distal true lumen of the CTO lesion.

We herein report the application of this technique for peripheral CTO lesions as an IVUS-guided reentry technique in EVT.

## Case presentations

### Case 1

**Fig. 1:**
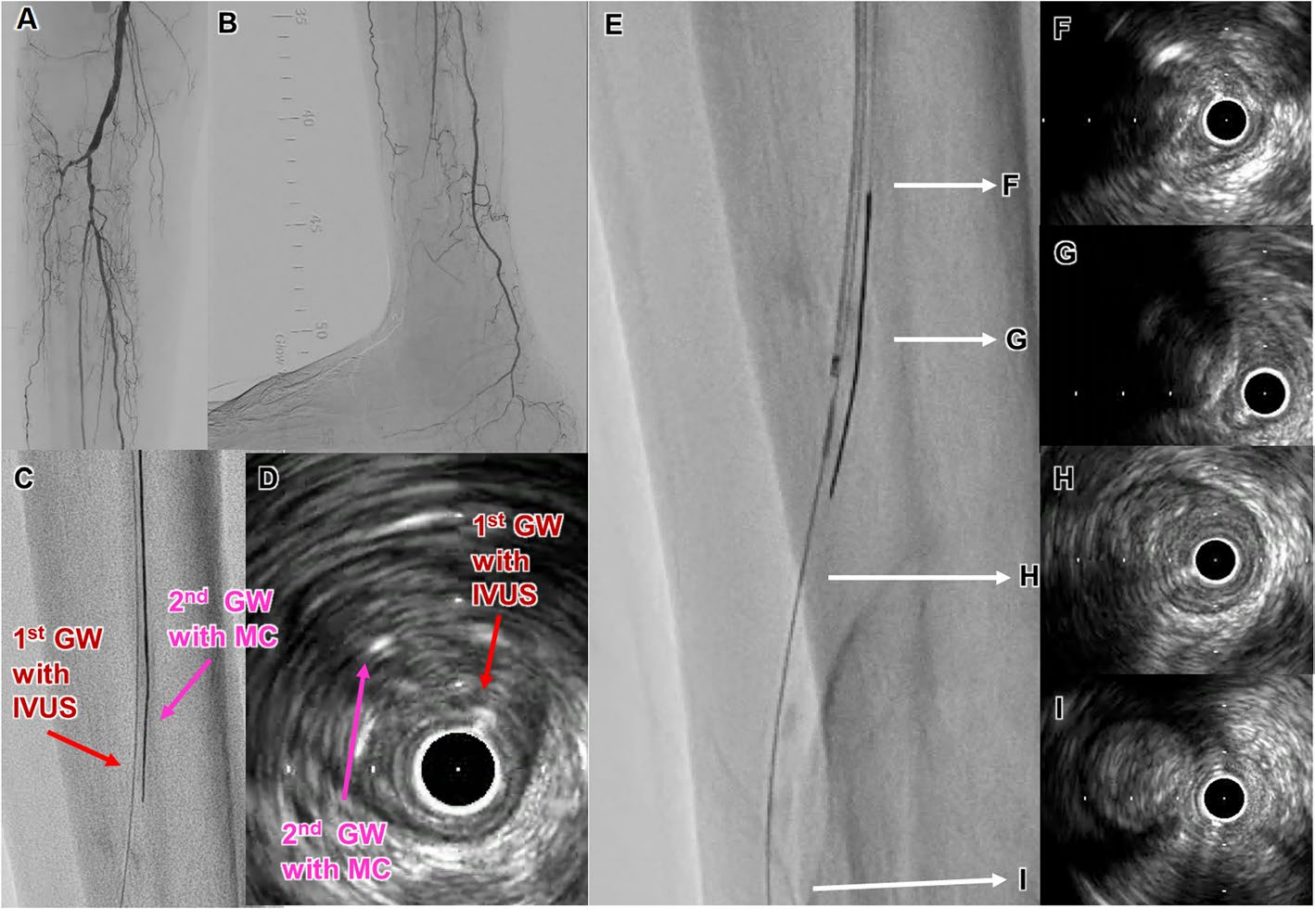
**A**,** B** Control angiography showed moderate to severe stenosis of the right popliteal artery, and total occlusion of the ATA to the DPA. **C** IVUS-guided parallel wiring was performed. The second GW with the Wingman MC could not be advanced because of severe calcification (red arrow: 1st GW with IVUS catheter, pink arrow: 2nd GW with Wingman MC). **D** IVUS findings of the CTO lesion in the ATA (red arrow: 1st GW with IVUS, pink arrow: second GW). **E** The second GW and IVUS were advanced in the SS from the mid-ATA. **F**,** G** IVUS findings of the CTO lesion in the ATA. The IVUS transducer was in the SS, and the true lumen was occupied by calcified plaque. **H** IVUS findings of the CTO lesion in the ATA. The IVUS transducer was in the widely expanded SS, and the true lumen was somewhat compressed and had become smaller. **I** IVUS findings of the CTO lesion in the distal ATA. The SS was relatively narrow, and the true lumen had little calcification, and it is relatively close to IVUS transducer

**Fig. 2:**
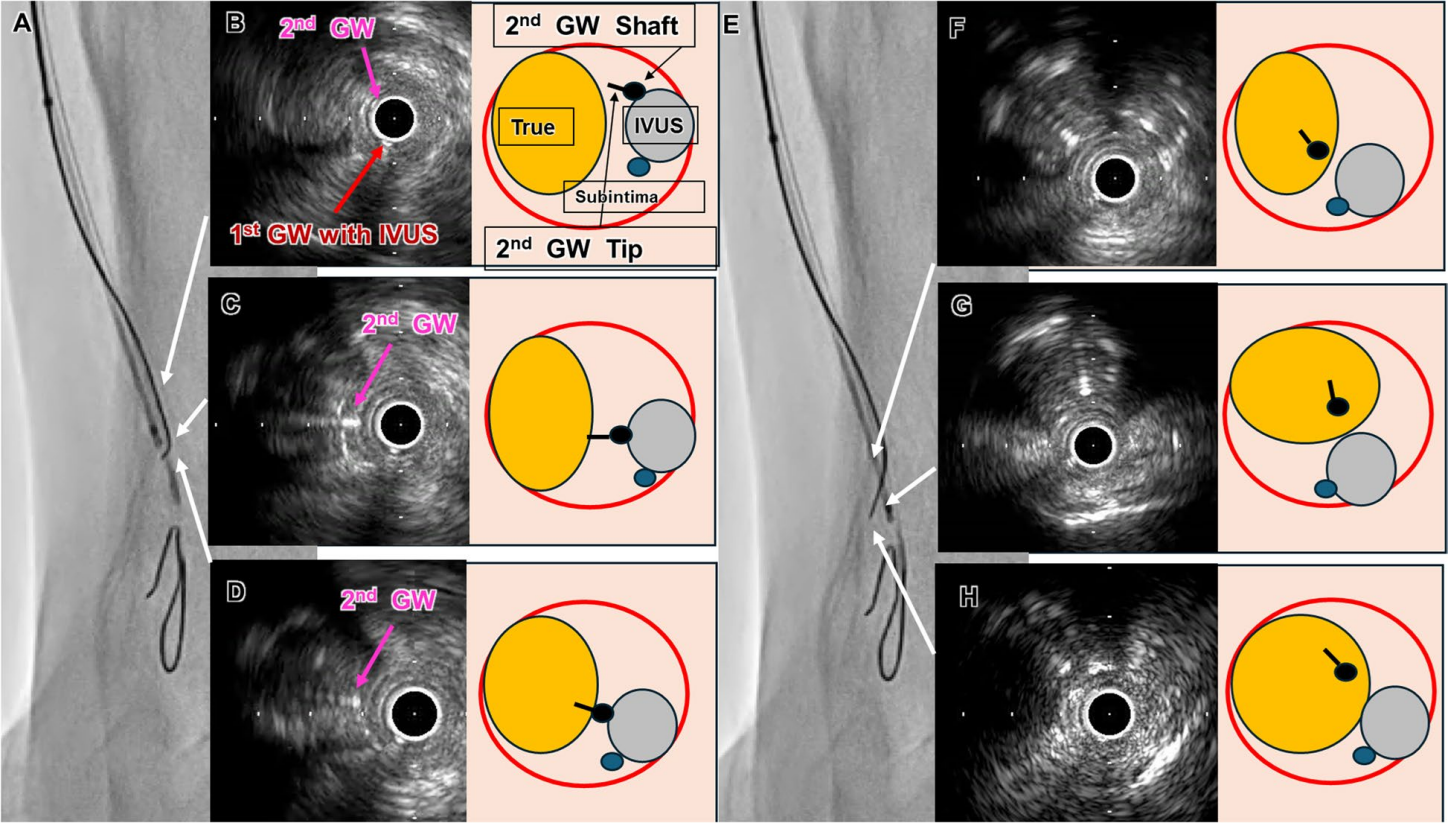
**A** The second GW was advanced from the SS to the intraplaque of the distal ATA using the TD method. **B** IVUS cross-sectional findings showing the start of reentry at the distal ATA (red arrow: 1st GW with IVUS, pink arrow: 2nd GW). We rotated it counterclockwise to point the tip in the direction of the occluded true lumen (intraplaque). **C** The GW tip was facing in the direction perpendicular to the true, so we advanced it in that direction. **D** We were able to confirm that the GW shaft and tip were going into the true, and we advanced while turning it slightly clockwise. **E** The second GW on fluoroscopy advanced from the right to the left of the IVUS catheter. **F** We were able to confirm that the GW shaft and tip were in the intraplaque, and we advanced while turning it slightly clockwise. **G**,** H** We advanced while turning it slightly counterclockwise

**Fig. 3:**
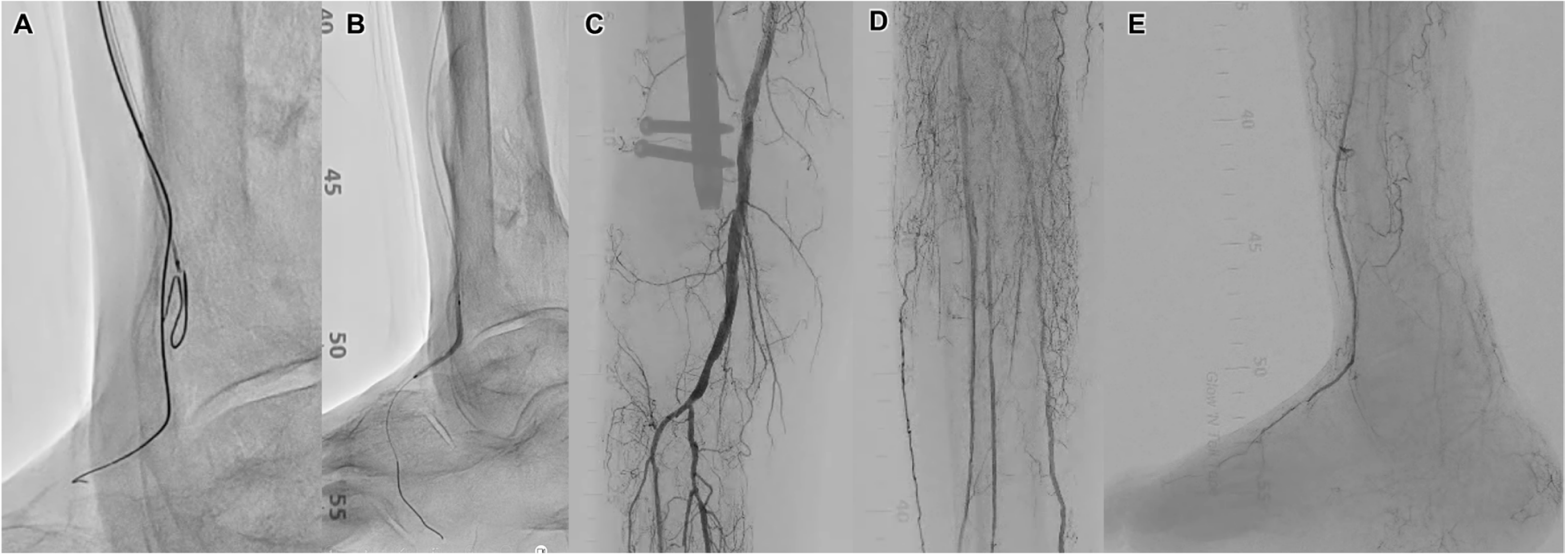
**A** The GW was advanced into the true lumen of the distal DPA. **B** The DPA was dilated with a 2- × 40-mm balloon. **C–E** Final angiography showed sufficient expansion of the target lesion and good antegrade flow

A 71-year-old man undergoing hemodialysis presented with ulceration in his right foot. A 5-Fr guiding sheath (Parent select5082^®^ guiding sheath; Medikit, Tokyo, Japan) was inserted into the right common femoral artery via the ipsilateral antegrade approach. Control angiography revealed stenosis in the popliteal artery and total occlusion of the anterior tibial artery (ATA) to the dorsalis pedis artery (DPA) with severe calcification (Fig. 1A, B). A 0.014-inch GW (Gladius^®^ GW; Asahi Intec, Aichi, Japan) and a microcatheter (Caravel^®^ microcatheter; Asahi Intec) seemed to advance into the subintimal space (SS) of the ATA CTO. (1)We then introduced the AnteOwl IVUS into the CTO, which confirmed that the GW was indeed in the SS from the proximal portion of the ATA CTO. (2) Then, using the IVUS-guided parallel wiring technique (EXCAVATOR technique), we advanced a 0.014-inch GW (Crosslead penetration^®^ GW; Asahi Intec) and microcatheter (Wingman 14 C^®^ microcatheter; Kaneka Medics, Tokyo, Japan) into the intraplaque (Fig. 1C, D), but the lesion was too difficult to cross because of severe calcification [5]. (3) We abandoned the intraplaque GW approach and proceeded with the GW and IVUS within the SS from the mid-ATA in order to find out the reentry point (Fig. 1E). IVUS revealed that while the mid-ATA had severe calcified plaque (Fig. 1F, G), the distal ATA was less calcified (Fig. 1H, I). The area where the SS is spreading (Fig. 1H) was not suitable as a reentry point, so we determined the distal ATA (Fig. 1I) seemed to be suitable for reentry (Fig. 1 ). (4) We introduced a second GW (ConQuest Pro12ST^®^ GW; Asahi Intec) and microcatheter (Ichibanyari PAD2^®^ microcatheter; Kaneka Medics) for preparing to perform the IVUS-guided reentry with TD. (5) We advanced the GW from the SS into the intraplaque of the distal ATA (Fig. 2A–H). The TD method allowed the GW tip to precisely penetrate the wall between the SS and the intraplaque in a nearly vertical direction (Fig. 2B–D). By closely observing the position of the GW shaft and the direction of the tip of GW using IVUS, the GW was advanced by rotating it clockwise or counterclockwise relative to the intraplaque (Fig. 2B-D, F-G). The GW was finally advanced into the true lumen of the distal DPA (Fig. 3A). The DPA was then dilated using a 2- × 40-mm balloon (Oceanus^®^; Medicos Hirata, Tokyo, Japan), the ATA with a 3- × 300-mm balloon (SHIDEN HP^®^; Kaneka Medics) (Fig. 2B), and the popliteal artery with a 6- × 100-mm drug-coated balloon (Ranger DCB^®^; Boston Scientific, Marlborough, MA, USA). Final angiography showed adequate expansion of the target lesion with good antegrade blood flow to the right toe (Fig. 2C–E). After EVT, the ulcer on the right toe healed without the need for additional intervention. 6 months after EVT, there was no recurrence of toe ulcers.

### Case 2

**Fig. 4:**
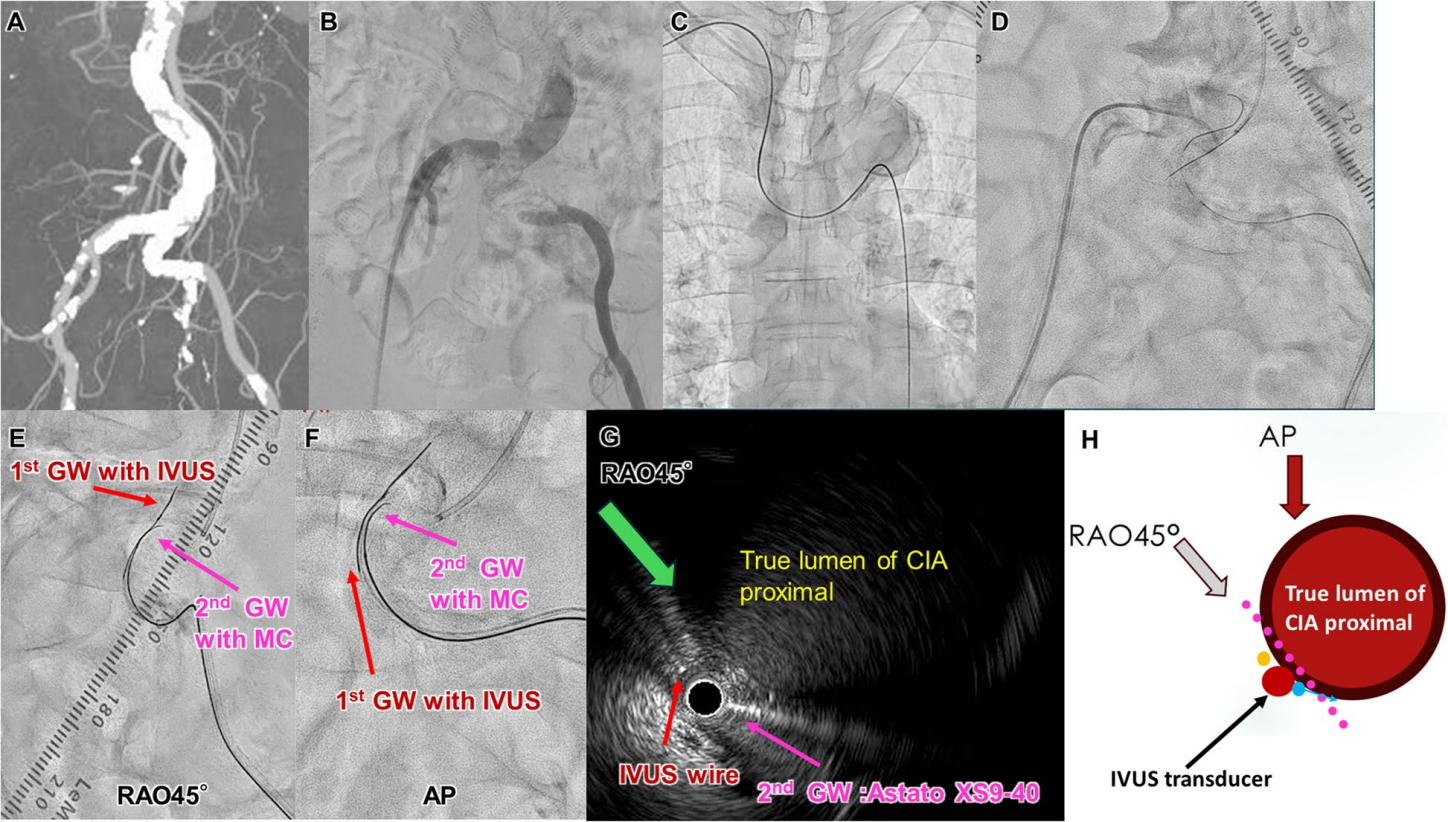
**A** Contrast-enhanced computed tomography and control angiography showed severe stenosis of the right CIA and occlusion of the left CIA. **C**,** D** Antegrade wiring was very difficult because of the tortuous descending aorta and left CIA. **E**,** F** Fluoroscopic images of right anterior oblique 45° and anteroposterior view, respectively (red arrow: first GW with IVUS, pink arrow: second GW with MC). **G**,** H** IVUS findings of proximal CIA. The IVUS transducer and second GW were in the SS (red arrow: IVUS on the wire, pink arrow: second GW). The green arrow indicates the right anterior oblique 45° view and the red arrow indicates the anteroposterior view

A 66-year-old man undergoing hemodialysis presented with severe intermittent claudication. Contrast-enhanced computed tomography and control angiography revealed severe stenosis of the right common iliac artery (CIA) and occlusion of the left CIA (Fig. 4A, B). EVT was initiated using a 7-Fr sheath in both femoral arteries, with antegrade wiring attempted from the right iliac artery and the right brachial artery (Fig. 4C, D). Despite this, antegrade wiring remained challenging because of the tortuosity of the descending aorta. The retrograde approach was also complicated by the flexion and calcification of the left CIA.

**Fig. 5:**
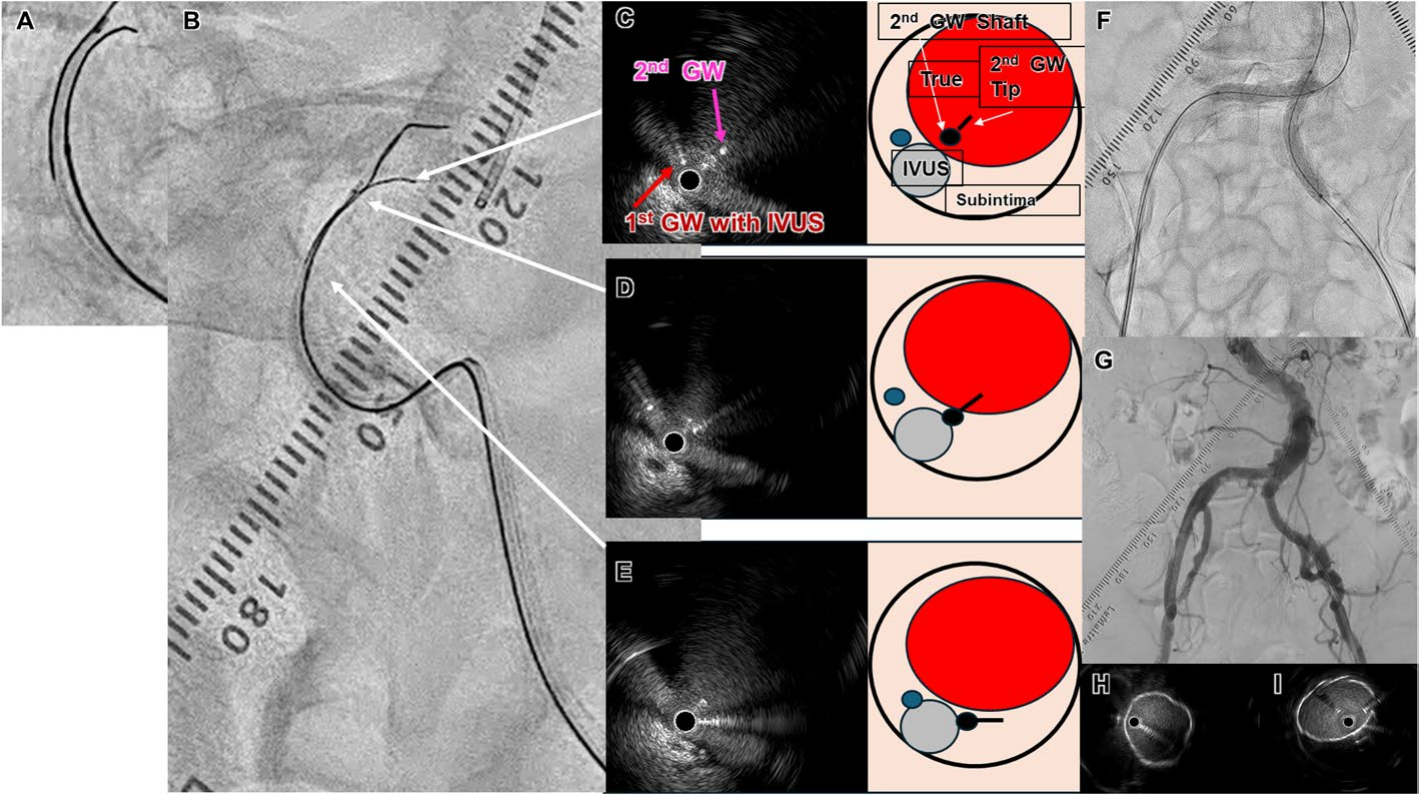
**A** The second GW with a large second curve. **B** The second GW was advanced toward the right side of the IVUS catheter in the right anterior oblique 45° view. **C–E** IVUS cross-sectional findings showing the start of reentry. The second GW was rotated in a counterclockwise direction from **E** to **C**, successfully achieving reentry from the SS to the true lumen in the proximal CIA (red arrow: first GW with IVUS, pink arrow: second GW). **F** Kissing stent graft implantation was performed. **G** Final angiography showed sufficient expansion of both CIA lesions with no perforation. **H**,** I** IVUS findings of each proximal CIA

(1) Subsequently, the AnteOwl IVUS was placed on the retrograde guidewire that had been advanced to the subintimal and inserted retrogradely via the left CFA, allowing visualization of the true lumen at the CIA entry. (2) First, the IVUS findings were aligned with the fluoroscopic images to enable three-dimensional IVUS-guided wiring, with the IVUS catheter being aimed toward the right side at a right anterior oblique angle of 45° for advancement (Fig. 4E–H). (3) The rotation of the tip of the second GW was then adjusted using the TD technique. A 0.014-inch GW (Astato XS9-40^®^ GW; Asahi Intec) with a large second curve (Fig. 5A) was advanced while rotating counterclockwise (Fig. 5E, D). This allowed successful reentry from the subintimal space to the true lumen in the proximal CIA (Fig. 5B–E).

Finally, two balloon-expandable stent grafts (Viabahn VBX^®^ stent graft; GORE, Newark, DE, USA) were implanted bilaterally as kissing stents—one on the right side (8.0 × 38 mm) and one on the left side (8.0 × 58 mm) (Fig. 5F). Final angiography and IVUS confirmed sufficient expansion of both CIA lesions (Fig. 5G–I). The patient’s claudication improved immediately postoperatively. 12 months after EVT, the ABI was 1.12 on the right side and 1.04 on the left side, and there was no evidence of restenosis on duplex ultrasound.

## Discussion

We reported successful revascularization for complex peripheral CTO using the IVUS-guided reentry technique with the TD method. To our knowledge, there are no reports applying this TD technique in EVT for peripheral artery disease.

**Fig. 6:**
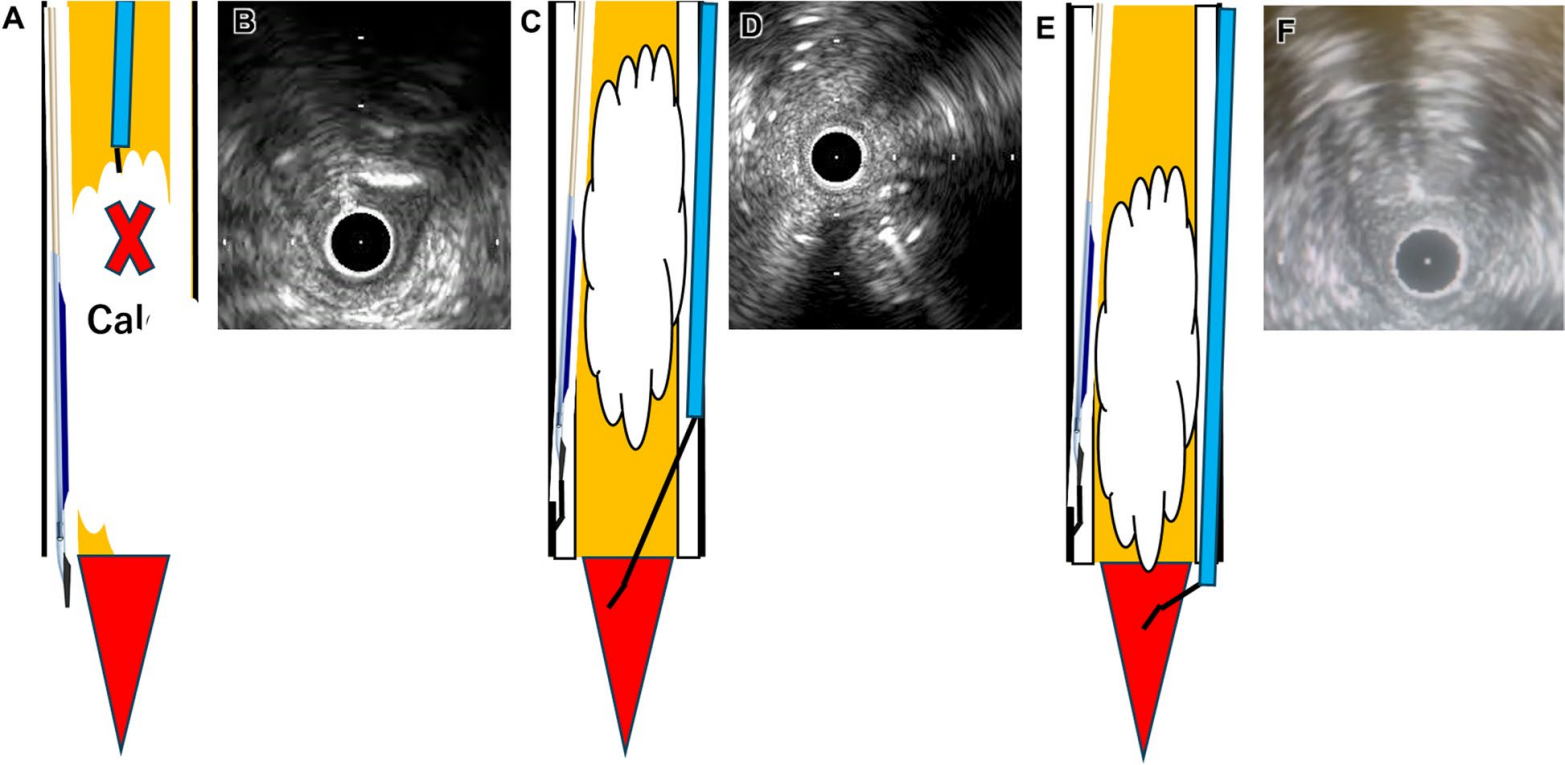
Comparison of the success and failure of IVUS-guided wiring for BTK CTO. **A**,** B** The IVUS device was able to pass through the SS, but the GW was unable to advance through the calcified plaque. **C**,** D** The second GW reentered the distal intraplaque, followed by advancement into the distal true lumen. **E**,** F** The second GW directly reentered the distal true lumen

The EXCAVATOR technique used three-dimensional IVUS-guided wiring [5]. However, the limitation of this technique is that while it indicates whether the GW should be advanced to the right or left of the IVUS catheter on the fluoroscopy screen, it does not show the direction in which the GW tip should be rotated. Additionally, there are cases in which severe calcification or tortuous vessels make it difficult to proceed with the intraplaque approach (Fig. 6A).

In Case 1, it was difficult to pass through the severely calcified intraplaque route of the middle ATA, and after diverting to the SS, reentry into the relatively unstiffened plaques of the distal ATA allowed the GW to pass into the true lumen of the distal DPA. If the distal true lumen is confirmed, as in TD-ADR in coronary CTO intervention, reentry at the distal true lumen can be performed in BTK CTO (Fig. 6C). However, in this case, since stent placement is not applicable in BTK CTO, reentry in the distal true lumen of the DPA may reduce the healthy distal lumen area due to hematoma. Therefore, we performed reentry in the intraplaque of the distal ATA and guided the GW into the distal true lumen for the final part (Fig. 6B).

In Case 2, the GW was advanced in the SS from the retrograde direction and reentered the true lumen of the left CIA ostium. In this case, the direction of the GW on fluoroscopy was clarified by reflecting the IVUS image onto the fluoroscopic view and fusing the two. By rotating the GW tip counterclockwise using the TD method, we successfully reentered the true lumen. This suggests that IVUS-guided reentry with TD may be beneficial not only for complex BTK CTO but also for other peripheral arteries where a bidirectional approach is challenging.

During IVUS-guided reentry, it is necessary to guide the GW from the SS into the true lumen, which requires precise manipulation of the GW while observing the direction of the tip and its position relative to the true lumen wall. Using TD, the GW tip can be manipulated in real time, enabling reproducible penetration into the target lumen.

The limitation of this technique is that it cannot be used for lesions that cannot be visualized with IVUS. Although the TD method using AnteOwl has been reported in PCI and theoretically could be feasible with other IVUS devices, it remains unclear whether this approach is practical in real-world peripheral CTO EVT. Further investigation is needed to confirm whether this method can be applied to peripheral CTO EVT. Nevertheless, it may offer a reproducible solution for challenging cases where GW passage is difficult.

## Conclusions

IVUS-guided reentry wiring with the TD technique may represent a breakthrough for GW crossing in complex peripheral CTO lesions.

## Supplementary Information

Supplementary Material 1.

Supplementary Material 2.

## Data Availability

The datasets used and/or analyzed during the current study are available from the corresponding author on reasonable request.

## Abbreviations

EVT: E ndovascular therapy
CTO: Chronic total occlusion
GW: Guidewire
BTK: Below-the-knee
IVUS: Intravascular ultrasound
EXCAVATOR: Extreme antegrade guidewire crossing by AnteOwl WR® intravascular ultrasound-guided parallel wiring to a below-the-knee artery
TD-ADR: Tip-detection antegrade dissection reentry
ATA: Anterior tibial artery
DPA: Dorsalis pedis artery
SS: Subintimal space
MC: Microcatheter
CIA: Common iliac artery
PCI: percutaneous coronary intervention
